# The Risk of Acrylamide Intake from Roasted Arabica Coffee (Pure, Torrefacto and Soluble) Consumed in Costa Rica

**DOI:** 10.3390/foods15122199

**Published:** 2026-06-18

**Authors:** Daniela Jaikel-Víquez, Ilhami Okur, Alejandra Gómez-Arrieta, Fabio Granados-Chinchilla, Graciela Artavia, Carolina Cortés-Herrera, Georgina Gómez-Salas, Mauricio Redondo-Solano, Bing Wang

**Affiliations:** 1 Departamento de Microbiología e Inmunología, Facultad de Microbiología, Universidad de Costa Rica, Ciudad Universitaria Rodrigo Facio, San José 11501-2060, Costa Rica; alejandra.gomez@ucr.ac.cr (A.G.-A.); mauricio.redondosolano@ucr.ac.cr (M.R.-S.); 2 Centro de Investigación en Enfermedades Tropicales (CIET), Universidad de Costa Rica, Ciudad Universitaria Rodrigo Facio, San José 11501-2060, Costa Rica; fabio.granados@ucr.ac.cr; 3 Department of Food Science and Technology, University of Nebraska-Lincoln, Lincoln, NE 68588, USA; iokur2@unl.edu (I.O.); bing.wang@unl.edu (B.W.); 4 Centro Nacional de Ciencia y Tecnología de Alimentos (CITA), Universidad de Costa Rica, Ciudad Universitaria Rodrigo Facio, San José 11501-2060, Costa Rica; graciela.artavia@ucr.ac.cr (G.A.); carolina.cortesherrera@ucr.ac.cr (C.C.-H.); 5 Escuela de Medicina, Universidad de Costa Rica, Ciudad Universitaria Rodrigo Facio, San José 11501-2060, Costa Rica; georgina.gomez@ucr.ac.cr; 6 Laboratorio de Investigación y Entrenamiento en Microbiología de Alimentos y Aguas (LIMA), Universidad de Costa Rica, Ciudad Universitaria Rodrigo Facio, San José 11501-2060, Costa Rica

**Keywords:** acrylamide, coffee, dietary exposure, GC-MS, risk assessment

## Abstract

Acrylamide (AA) is a contaminant with carcinogenic and genotoxic properties that occur in heat-produced food products. This study aimed to evaluate the occurrence of AA in different coffee products commercially sold in retail markets of Costa Rica and to develop a probabilistic exposure assessment model to assess the potential human health risk due to its consumption. The average AA concentration in the coffee samples analyzed (*n* = 110) was 110.29 ± 151.61 µg kg^−1^. The mean dietary exposure (DE) values, for the middle-bound (MB) approach, varied from 0.025 to 0.083 µg kg^−1^ BW per day. The margin of exposure (MOE) was calculated with a BDML_10_: 430 μg kg^−1^ BW day^−1^ for neurotoxicity and 170 μg kg^−1^ BW day^−1^ for cancer effect, according to EFSA (2015). No neurotoxicity risk was identified as MOE values ranged from 4291 to 467,984 for the adult male population, from 4566 to 477,203 for the adult females, from 4265 to 506,062 for the male minors and from 2512 to 495,151 for the female minors. On the other hand, MOE values for the carcinogenic risk were below 10,000 for the mean and P95th coffee consumers, denoting a possible health concern. The values ranged from 1696 to 6717 for the adult male population, from 1805 to 7201 for the female adults, from 1686 to 6304 for the male minors and from 993 to 2155 for the female minors. The mean incremental lifetime cancer risk (ILCR) values for male adult, female adult, male minor, and female minor were 1.7 × 10^−5^, 1.6 × 10^−5^, 1.9 × 10^−5^, and 3.9 × 10^−5^, respectively, for the MB approach. These results denote a potential or considerable risk in consumption of coffee due to AA intake. Thus, no neurotoxicity risk was identified; however, a potential carcinogenic risk was observed based on MOE and ILCR results.

## 1. Introduction

Acrylamide (AA) is a low molecular weight, organic compound that is formed during the Maillard reaction as a byproduct of the decarboxylation and deamination of asparagine in the presence of reducing sugars (glucose and fructose), at temperatures higher than 120 °C [1,2,3], and low moisture levels [4]. Therefore, AA is a thermal processing contaminant formed in many common carbohydrate-rich foods during cooking processes like baking, frying, and roasting [1,2]. It is a neurotoxic, carcinogenic, and genotoxic compound [3] that was classified as a Group 2A probable carcinogen by the International Agency for Research on Cancer (IARC) in 1994 [5]. As for the mechanism of action, after ingestion, AA is rapidly absorbed in the gastrointestinal tract and translocated to all tissues and organs [6]. In the liver, for example, AA is oxidized to a reactive epoxide named glycidamide via the cytochrome P450 2E1 (CYP2E1) enzymatic system [2,6]. Glycidamide has been reported to be a potent mutagen and clastogen. It causes guanine:cytosine (G:C) to thymine:adenine (T:A) transversions; damages the brain stem, spinal cord, and peripheral neurons; and causes thyroid, mammary gland, and pancreatic islet tumors [2].

As previously stated, AA is found in commonly consumed foods like baked (cookies, breads, potatoes, pastries), fried (potatoes, vegetable crisps), toasted goods (cocoa, coffee, nuts, bread), cereals (breakfast cereals, pasta) and baby foods (biscuits and formula) [4,7]. In the specific case of coffee, the roasting process usually takes place at 200 °C for less than 20 min [3]. This process involves dehydration of the product and roasting, where the Maillard and Strecker reactions occur, giving it its characteristic aroma and color [3]. Unfortunately, the roasting temperatures favor the production of AA, which is highly soluble in water and thus easily transferred from the coffee powder to the beverage during the brewing process [8]. Also, the percentage of extraction is increased as the volume of water used to prepare coffee increases [9]. It is worth noting that differences in the levels of AA have been reported after the roasting of the beans that come from the different coffee plant species. For example, *Coffea canephora* var. Robusta’s roasted beans usually present higher levels of AA than *Coffea arabica*’s [3,9].

As previously stated, AA levels have been reported in many food products. In the case of coffee, AA concentrations ranged from 98 to 298 µg kg^−1^ in Spain, 185 to 293 µg kg^−1^ in Italy [4], and 133 to 549 µg kg^−1^ in Iran [6]. Therefore, on 20 November 2017, the European Commission (2017/2158/EU) published the document “Establishing, mitigation, measures and benchmark levels for the reduction of the presence of acrylamide in food”, where they established a maximum permitted level (MPL) of 400 µg kg^−1^ for roasted coffee and 850 µg kg^−1^ for instant coffee [2].

Costa Rica is a Central American country famous for its gourmet-quality Arabica coffee. According to the statistic from the Costa Rican Institute of Coffee (ICAFE, from the Spanish *Instituto del Café*), in the harvest 2024–2025, Costa Rica produced 1,350,000 60 kg bags of coffee [10] and is the second largest coffee consumer in Latin America ([4.2–4.3] kg per capita per year) [11], so it is crucial to determine the daily intake and risk of AA consumption of the coffee commercially sold in this country. Thus, the objectives of this study were to evaluate the occurrence of AA in coffee products commercially sold in retail markets of Costa Rica and to develop a probabilistic exposure assessment model to assess the potential human health risk of AA exposure among coffee consumers in this country.

## 2. Materials and Methods

### 2.1. Coffee Consumption

The coffee consumption patterns of the Costa Rican urban population were obtained from the Latin American Study of Nutrition and Health (ELANS, from the Spanish *Estudio Latinoamericano de Nutrición y Salud*). ELANS is a household-based multi-national cross-sectional survey that collected food intake information through two nonconsecutive 24 h day dietary recalls. The interviews were conducted in person from March 2014 to December 2015 [12,13]. In the case of Costa Rica, there were 793 participants enrolled, out of which 595 were coffee consumers. The information retrieved was sex, age, body weight (kg), daily coffee consumption (g), coffee types consumed (pure roasted, Torrefacto, and soluble) and coffee brands. The participants were divided into four subpopulations: male adults, female adults, male minors and female minors. The minor subpopulations comprised participants who ranged from 15 to 17 years old. These were the youngest participants of the ELANS survey; even though this is a narrow age range, compared to other studies that take into consideration younger children, it is worth noting that coffee is a beverage mainly consumed by grown-ups and adolescents, and not by toddlers or small children. It is worth noting that the ELANS survey is the most recent nutritional survey conducted in Costa Rica to date.

### 2.2. Occurrence of Acrylamide in Coffee Samples from Costa Rica

#### 2.2.1. Reagents

An analytical standard for acrylamide (catalog number 23701) and deuterated acrylamide-2,3,3-d3 (catalog number 636568, 98 atoms % D, D2C=CDCONH2, used as internal standard) were used during chromatographic analysis. Magnesium oxide (catalog number 243388), acetonitrile (ACN, gradient grade, ≥99.9%, catalog number 439134), sodium chloride (ACS reagent, ≥99.0%, catalog number S9888), and sodium sulfate (anhydrous, ACS reagent, powder, catalog number, 238597) were purchased from Millipore Sigma (Saint Louis, MO, USA). Ultrapure water (type I, 0.055 µS cm^−1^ at 25 °C, 5 µg L^−1^ TOC) was obtained using an A10 Milli-Q^®^ Advantage system and an Elix^®^ Advantage 10 system (Merck KGaA, Darmstadt, Germany). A quality control material of known AA concentration for ground coffee was used to assess method accuracy (product code FCCP3-DRH10QC and purchased from ISO/IEC 17043/2010 accredited FAPAS^®^, Sand Hutton, York, UK).

#### 2.2.2. Samples

A survey following the EC 401/2006 guidelines [14] was conducted from November 2020 to December 2021 to obtain Costa Rican, commercial, Arabica, roasted, grounded coffee samples. The sample size was determined by applying Cochran’s formula (Equation (1)) [15].

(1) $$n=\frac{Z^{2}pq}{e^{2}}$$

where *n* is the sample size; e is the desired level of precision; *p* is the estimated proportion of the population to which the question is attributed; q is 1 − *p;* and z is the z-value extracted from a z-table (for a 95% confidence level and a ± 5% precision, the value for z is 1.96). As a result, a total of *n* = 110 roasted Arabica grounded coffee packages (pure roasted [*n* = 86], Torrefacto or sugar-added roasted coffee [*n* = 12], and soluble coffee [*n* = 12]) were purchased. The packages were from 26 different brands and different batches. It is important to specify that brand selection was representative of the general consumption habits of the Costa Rican urban population (according to the ELANS survey) and according to market availability. They were purchased in various supermarkets located in the provinces of San José, Heredia, Alajuela and Cartago (Great Metropolitan Area of Costa Rica). The pure roasted coffee samples were also sorted by their roasting process into light roast (*n* = 7), medium roast (*n* = 49), and dark roast (*n* = 30). The samples were stored at 6.1 °C until the experiments were performed.

#### 2.2.3. AA Extraction from Coffee Samples

Three grams of the previously homogenized coffee samples were weighed in a centrifuge tube (50 mL, polypropylene, conical bottom, Corning^®^, Corning, NY, USA) and 2 g of magnesium oxide was added. Afterward, 15 mL of water was added to the mixture, which was then shaken for 5 min using a vortex (SI™ Vortex-Genie™ 2, Scientific Industries Inc., Bohemia, NY, USA) and incubated in a water bath (Precision™ CIR 35, Thermo Scientific™, Waltham, MA, USA) at 70 °C for 40 min. The blend was left undisturbed until it reached room temperature and then centrifuged for 10 min at a RCF of 4696× *g* (catalog 75004241, Sorvall™ ST 16R Series, Thermo Scientific™, MA, USA).

The recovered supernatant was mixed with 10 mL of ACN and 100 µL of a 50 µg mL^−1^ solution of the internal standard. For each batch of coffee quantified, a spiked sample was used as additional quality control, which was treated as above but also included 150 µL of a 20 µg AA mL^−1^ solution. The resulting mixture was stirred using an orbital shaker at 200 rpm for 30 min (KS 130 Basic, IKA™, Staufen, Germany) and was then appended with 4 g sodium sulfate and 0.5 g sodium chloride and stirred for an additional 10 min. The mix was centrifuged at 4 °C for 8 min. A 1 mL aliquot of the supernatant was finally recovered and passed through a syringe filter (0.20 µm, regenerated cellulose, catalog 18407, Sartorius, Gotinga, Germany) into a GC/MS-ready vial (2 mL vials and 11 mm aluminum crimp vial caps, red silicone/clear PTFE seal, catalog number 5190–9045, Agilent Technologies, Santa Clara, CA, USA) [16].

#### 2.2.4. AA Chromatographic Separation and Quantification

**Figure 1:**
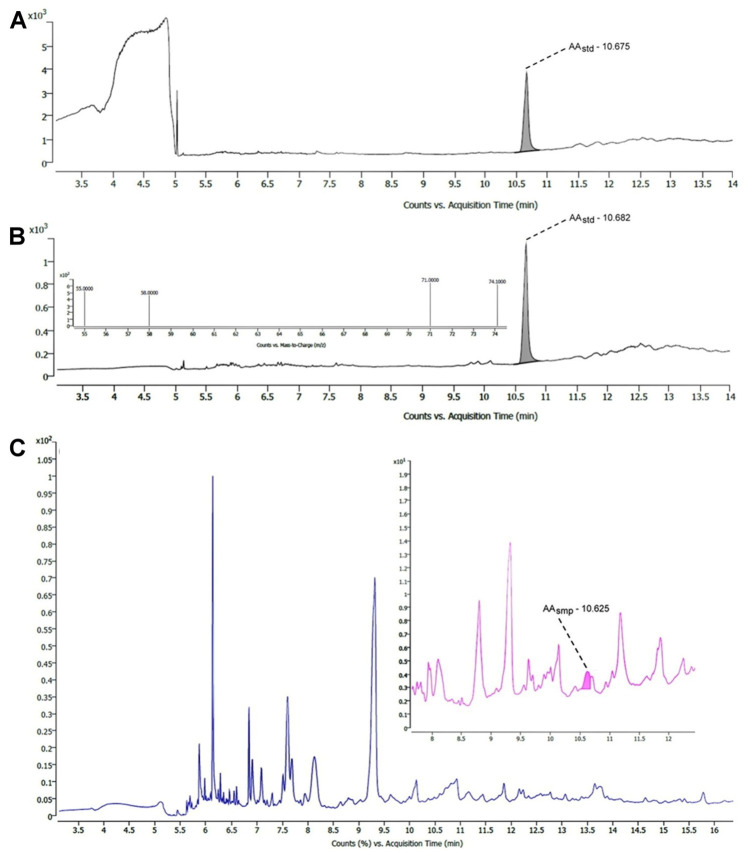
Chromatographic analysis for AA in coffee samples, where (**A**) is a Total Ion Chromatograph (TIC) of a ca. 80 µg mL^−1^ AA standard in ACN, point 7th of the 9-point calibration curve; (**B**) Extracted Ion Chromatogram (EIC_71 m/z_) for the standard. Overlay: the selected ions of 55, 58, 71, and 74 m/z used for monitoring the AA molecule are shown. (**C**) TIC for a roasted pure coffee sample coded M67, which tested positive for AA. Overlay: EIC_71m/z_ obtained for the same sample (M67), only the region of interest (covering the elution time for AA) is shown.

For the quantitative analysis, an Agilent 7820A gas chromatograph coupled with a 5977B single quadrupole (SQ) mass spectrometry detector and equipped with a 30 m × 250 μm × 0.25 μm chromatographic column (catalog 122-7032UI, J&W DB-WAX Ultra Inert, Agilent Technologies, CA, USA) was used, injection volume was set to 5 μL in splitless mode at 15 mL min^−1^, 150 °C, and a helium flow of 1 mL min^−1^ (220 cubic ft, ultra-pure grade, GasPro, San Antonio, Alajuela, Costa Rica), was used. To separate AA, a temperature ramp of 60 °C for 4 min, 70 °C min^−1^ to 160 °C for 4 min, and 10 °C min^−1^ to 190 °C was applied. This temperature was sustained for four more minutes, totaling 17 min of analysis. The detection parameters were: ionization was mediated by an electron impact system at 70 eV, and the quadrupole temperature was set at 280 °C; selected ions of 55, 58, 71 y 74 m/z were observed using SIM mode (Simultaneous Ion Monitoring). The retention time (analyte signal) was recorded under these conditions at 10.682 ± 0.225 min (Figure 1). The internal standard was assessed in all samples and standards at a fixed final concentration of 1.5 µg mL^−1^, and nine-point calibration curves in ACN were constructed from 0.1 to 100 µg mL^−1^ during each measurement and used to interpolate sample data [16]. The limits of detection (LOD) and quantification (LOQ) were 25.00 and 75.75 µg kg^−1^, respectively. Table 1 summarizes the validation parameters. It is important to note that single quadrupole mass spectrometers are used for routine compound screening, purity analysis, and reliable quantification. Additionally, quantification using SQ GC-MS was improved using stable isotope-labeled analogs (i.e., *d*_3_-acrylamide) as an internal standard.

#### 2.2.5. Statistical Analysis

The statistical analysis was conducted with the program SPSS^®^ (IBM^®^ SPSS^®^ Statistics software for Windows, version 20.0. Armonk, NY, USA). The arithmetic mean, standard deviation, and interval of body weight (BW) and coffee consumption were determined for each subpopulation (male and female [total, adults, and minors]). The percentage of coffee consumers was also determined for each subpopulation. The arithmetic mean, standard deviation, median, minimum, and maximum concentrations for AA contamination were determined for the different types of coffee. Finally, an ANOVA test coupled to a Tukey post hoc test was performed to determine if there were any differences between the AA concentration of the different roasting processes.

#### 2.2.6. Dietary Exposure Estimation

AA dietary exposure (DE) due to consumption of coffee in Costa Rica was estimated according to the following Equation (2) [17], and the results were expressed as micrograms per kilogram of BW per day for each subpopulation:

(2) $$DE=\frac{IR\times C}{BW}$$

where IR is the daily intake rate (g day^−1^) of coffee, C is the AA concentration in coffee (µg kg^−1^), and BW is the body weight for each population group (kg). It is worth noting that the AA concentrations obtained resemble those of the transferred metabolite into the coffee beverage, since the extraction procedures were performed with water at 70 °C.

Male adults, female adults, male minors, and female minors were the subpopulation groups targeted in calculating DE values. Based on these subpopulations, a probabilistic approach was used to calculate IR and BW values, and the best fit was found by using @Risk^®^ 8.5 (Palisade Corp., Ithaca, NY, USA). Regarding the concentration data, a probabilistic approach was used to find the distribution of AA concentration in coffee. For the AA concentration data below the LOD, three different scenarios were used [18]. Data below LOD was converted to 0 for a lower bound (LB) approach, to LOD/2 for a middle bound (MB), and to LOD for an upper bound (UB). For DE calculations for each subpopulation and coffee type, Monte Carlo simulation with Latin Hypercube sampling was performed with 50,000 iterations using @Risk^®^ 8.5 (Palisade Corp., Ithaca, NY, USA).

### 2.3. Risk Characterization

#### 2.3.1. Margin of Exposure (MOE)

To estimate the non-cancer (neurotoxicity) and cancer (carcinogenicity and genotoxicity) risk related to AA DE, the margin of exposure (MOE) approach was applied. MOE values were calculated by dividing the benchmark lower dose level (BDML_10_) by the AA DE [4]. For neurotoxicity, a BDML_10_ value of 430 μg kg^−1^ BW day^−1^ was used, and for carcinogenicity risk, 170 μg kg^−1^ BW day^−1^ [19]. For MOE calculations, Monte Carlo simulations with Latin Hypercube sampling were conducted with 50,000 iterations using @Risk^®^ 8.5 (Palisade Corp., Ithaca, NY, USA).

#### 2.3.2. Incremental Lifetime Cancer Risk (ILCR)

The incremental lifetime cancer risk (ILCR) was proposed by the Environmental Protection Agency (EPA) related to AA dietary exposure, and it was calculated according to the following Equation (3):

(3) $$ILCR=\frac{C\times IR\times ED\times EF\times SF\times CF\times ADAF}{BW\times AT}$$

where C is the acrylamide concentration in coffee (μg kg^−1^), IR is the coffee consumption for different groups (g day^−1^), BW is the body weight for each population group (kg), ED is the exposure duration (43 years for male adults, male minors, female adults and female minors), EF is the exposure frequency (365 days year^−1^), SF is slope factor (0.5 mg kg^−1^ day^−1^), CF is the correction factor (10^−6^ mg μg^−1^), ADAF is the adjustment factor (1 is for male adults, male minors, female adults and female minors) and AT is the lifetime period over which exposure is averaged for carcinogens (25,550 days). Monte Carlo simulation with Latin Hypercube sampling was carried out in 50,000 iterations using @Risk^®^ 8.5 (Palisade Corp., Ithaca, NY, USA) for ILCR calculation.

## 3. Results

### 3.1. Coffee Consumption Data

Five hundred and ninety-five out of the 793 (75.0%) Costa Rican participants from the ELANS survey reported coffee consumption. As previously stated, the participants were distributed into four subpopulations: male adults, female adults, male minors and female minors. The adult male population had a mean BW of 77.72 kg and consumed 17.81 g of coffee daily. The adult female population weighed 70.54 kg and consumed 15.25 g of coffee. The male minors had a mean BW of 69.53 kg and consumed 13.82 g of coffee. And finally, the female minor had a BW of 58.51 kg and consumed 15.52 g. As for types of coffee preferred, 60.9% of the male adult population consumed pure roasted coffee, 36.9% Torrefacto and 2.2% soluble coffee. Sixty-six percent of the female adults consumed pure roasted coffee, 31.2% Torrefacto and 2.0% soluble coffee. As for the minor population, the boys drank pure roasted coffee and Torrefacto in equal proportions, while the girls preferred pure roasted coffee (73.3%) over Torrefacto (26.7%). None of the adolescent populations referred to consuming soluble coffee.

### 3.2. Occurrence of AA in Coffee Samples from Costa Rica

A total of *n* = 110 coffee samples were analyzed, out of which 56.4% (*n* = 62) were contaminated with AA. The mean concentration for all the samples analyzed (above LOD and below LOD) was 110.29 ± 151.61 µg kg^−1^. Soluble coffee presented the highest AA concentration (255.74 ± 233.14 µg kg^−1^), followed by pure roasted coffee (105.38 ± 129.91 µg kg^−1^). It is worth noting that when calculating the mean values for all the samples, high SD values were found. This is due to the heterogenicity of the samples because more than half of them presented levels of AA below LOD and those positive for the chemical ranged from 33.41 to 759.59 µg kg^−1^. None of the Torrefacto samples presented AA levels above LOD. It is worth noting that three of the pure roasted coffee samples presented levels of AA that were above the LOD but below the LOQ, and four exceeded the MPL of 400 µg kg^−1^ for roasted coffee, with values that ranged from 446.14 to 696.18 µg kg^−1^. None of the soluble coffee samples exceeded the MPL of 850 µg kg^−1^ established for this product [2]. Also, according to the roasting conditions, the dark roast pure coffee presented the highest mean concentration (140.87 ± 175.73 µg kg^−1^), followed by the light roast (137.44 ± 150.43 µg kg^−1^) and lastly, the medium roast (79.07 ± 84.29 µg kg^−1^). No statistical differences were found between the AA concentration of the different roasting conditions (F = 2.385; df = 2; *p* = 0.098). Table 2 shows the concentration and occurrence of AA in the positive samples or samples with AA levels above the LOD.

### 3.3. Dietary Exposure Estimation (DE)

The mean, 5th, 50th and 95th percentiles of the estimated DE to AA for each subpopulation are presented in Table 3. Based on the different scenarios, the lowest mean DE was observed for the female adults with 0.024 µg kg^−1^ BW per day, under the lower bound approach, whereas the highest mean DE was found for the female minors with 0.087 µg kg^−1^ BW per day, at the upper bound approach. As for the middle-bound scenario, the mean DE was as follows: 0.025 µg kg^−1^ BW per day for female adults, 0.027 µg kg^−1^ BW per day for male adults, 0.028 µg kg^−1^ BW per day for male minors, and 0.083 µg kg^−1^ BW per day for female minors.

### 3.4. Risk Characterization

#### 3.4.1. Margin of Exposure (MOE)

Neurotoxicity is considered a non-neoplastic outcome; therefore, MOE values above 100 are accepted as a safety margin for no health concern. On the other hand, when carcinogenic or genotoxic effects are being studied, MOE values below 10,000 are considered of concern [20]. As shown in Table 4A,B, the MOE values for different populations and scenarios ranged between 2512 and 506,062 for neurotoxicity and 993 and 200,071 for carcinogenicity. The estimated values for MOE neurotoxicity were higher than the safety limit for all populations in all cases tested. Therefore, it can be said that there is no neurotoxicity risk of AA daily exposure due to the consumption of grounded, roasted, Arabica coffee commercially sold in Costa Rica. Regarding neoplastic effects, MOE values were higher than 10,000 for the 5th and 50th percentile coffee consumers, but lower than this limit for the mean and 95th percentile coffee drinkers. The lowest MOE values were found for the female minors, since they had a lower BW and consumed a large amount of coffee. These two factors result in larger DEs and, thus, in lower MOE values. Also, they consumed the lowest amount of Torrefacto coffee, which had non-detectable levels of AA in the samples tested.

#### 3.4.2. Incremental Lifetime Cancer Risk (ILCR)

The ILCR was estimated to evaluate the possibility of human cancer throughout life. The cumulative probability ILCR results for different populations in Costa Rica are shown in Table 5. The mean ILCR values for male adult, female adult, male minor, and female minor were 1.7 × 10^−5^, 1.6 × 10^−5^, 1.9 × 10^−5^, and 3.9 × 10^−5^, respectively, for the MB approach. According to Arabameri et al. (2026), ILCR values lower than 10^−6^ indicate that there is an acceptable or insignificant risk, and ILCR values higher than 10^−4^ indicate a serious health risk [21]. Based on the results obtained, it can be said that there is a potential carcinogenic risk for the mean and 95th percentile coffee consumers from the urban regions of Costa Rica.

## 4. Discussion

In the present study, almost 60% of the samples analyzed were contaminated with AA. This percentage of contamination is lower than those reported in other countries. For example, in a study conducted in Poland and another in Iran, AA was detected in all the samples tested [6,22]. As for the levels of AA in the different coffee types, the Costa Rican pure roasted coffee had an average concentration of 105.36 ± 129.91 µg kg^−1^, which is lower than that found in coffee sold in Spain (194 µg kg^−1^) [4], Poland (222.3 µg kg^−1^) [22], Italy (228 µg kg^−1^) [4], the United States of America (272.4 µg kg^−1^) [23] and Iran (549 µg kg^−1^) [6]. The levels of AA in soluble coffee sold in Costa Rican retail markets (255.74 ± 233.14 µg kg^−1^) were lower than those in the Ethiopian samples (421 µg kg^−1^) [24], but higher than those in the Polish ones (166.7 µg kg^−1^) [22]. However, as with roasted pure coffee, the prevalence was almost half in the Costa Rican samples.

Numerous factors may affect AA levels in foods, for example, the concentration of asparagine and reducing sugars, the pH, and the water activity [8,23]. In the specific case of coffee, as previously stated, *Coffea canephora* var. Robusta has more asparagine and fructose than *Coffea arabica*, so it usually presents higher levels of AA [3,9]. This could explain the lower concentrations of AA found in Costa Rican coffee since, by law, only *C. arabica* var. Caturra and Catuaí could be cultivated [25]. Also, immature coffee beans have more asparagine [26]. In Costa Rica, coffee is manually collected and according to Article 15 of the Costa Rican Law N°. 9872 from 28July 2020, the processing plants can receive up to 2% of immature coffee beans, ensuring a high-quality product that would have less asparagine, which is the limiting factor in the formation of AA. No AA was detected in the Torrefacto samples. Torrefacto is a type of roasted coffee that has up to 15 g/100 g of sucrose or glucose added during the roasting process [27,28]. Since the sugar content is higher for this product, it could be assumed that the levels of AA would be higher. However, it has been reported that the Maillard reaction between asparagine and glucose occurs at a higher rate in equimolar model systems as compared to systems with excess sugar. Also, in systems with excess sugars, these compounds are also consumed in caramelization reactions [29].

On the other hand, AA is produced in the early stages of the roasting process since free amino acids and reducing sugars are rapidly consumed in the Maillard reaction [8]. Then, its level decreases because of thermochemical degradation. Therefore, lighter roasting usually presents higher levels of AA than the dark roasting counterparts [3,9]. However, we did not find significant statistical differences among the samples analyzed (*p* > 0.05); however, the amount of light-roasted samples analyzed was small because the sampling was conducted based on consumer preferences obtained in the ELANS survey, and light-roast coffee is not usually consumed in Costa Rica. Thus, further analysis, with larger sample numbers, is required to determine possible differences between the roasting processes.

Previously, DE to AA for potatoes, plantains and cassava crisps, consumed in Costa Rica, were published [16]. However, to our knowledge, this study is the first probabilistic report to estimate the DE and risk associated with AA for the Costa Rican urban population due to coffee consumption. Overall, DE values in this study varied from 0.001 to 0.153 µg kg^−1^ BW per day. In European countries like Belgium, Denmark, Finland, France, Germany, Hungary, Ireland, Italy, Latvia, Spain, and Sweden, the DE values for roasted coffee beverage consumption are similar to ours (0.003 and 0.171 µg kg^−1^ BW per day) [20,30,31,32]. The same can be said for the DE reported in different regions of Ethiopia, where Sidama and Nekemte coffee are drunk [33]. For soluble coffee consumed in Spain, the median DE value was 6.8 ± 1.0 ng kg^−1^ BW per day [34]. Basaran and Aydin (2020) stated that the mean value for AA exposure in Turkey was between 0.09 and 0.17 µg kg^−1^ BW per day [35]. Also, in Turkey, a study found a DE value of 0.01 ± 0.03 µg kg^−1^ BW per day for pregnant women [17]. El-Zakhem Naous et al. (2018) found that dietary exposure due to consumption of American coffee in Lebanon was 0.37 ± 0.24 µg kg^−1^ BW per day. However, this value was increased to 10.9 ± 0.24 µg kg^−1^ BW per day for the consumption of Lebanese coffee [36]. Based on these findings, DE to AA in Costa Rica was parallel with the literature. However, the DE is lower in countries like Japan [37] and Iran [38]. Kawahara et al. (2018) indicated that DE to AA due to the consumption of brewed and powder coffee was 11 and 9 ng kg^−1^ BW per day in Japan, respectively [37]. And Karami et al. (2022) reported DE values for instant and pure coffee of 0.845 and 0.815 ng kg^−1^ BW per day, respectively [38]. In the case of the latter, AA concentration found in coffee was similar to our findings, but coffee consumption caused this difference. In the paper, it was mentioned that the daily coffee consumption was around 0.175 g per person [38], while coffee consumption in Costa Rica was roughly 110-fold higher.

The MOE approach characterizes the potential for human health associated with the contaminants’ presence in food. There are some uncertainties about this approach of AA in food since there is a lack of data on human internal and external exposure. Even though there are some uncertainties, this approach is commonly used for risk characterization concerning AA in foods. Based on our results, it was found that there was a potential carcinogenic risk, but no neurotoxicity risk. Claeys et al. (2016) found that there were neoplastic effects in Belgium due to the consumption of coffee in adults and minors based on their MOE results, as well [20]. Similar results were also found in Denmark, France, and Sweden [32,39,40]. Karami et al. (2022) reported neither neurotoxicity nor carcinogenic risk in the consumption of coffee in Iran. As previously explained, this is due to a consumption of 0.175 g per day, a much lower quantity than that reported by the other countries [38]. For Latin American countries like Brazil, Colombia, Mexico, and Peru, Guadalupe et al. (2024) indicated that there were nongenotoxic and genotoxic effects due to the consumption of instant coffee [41].

The results of our study showed that the mean ILCR values of AA for different urban populations in Costa Rica due to coffee consumption were between 4.3 × 10^−7^ (LB for male minors) and 1.5 × 10^−4^ for UB female minors. Even for the participants who reported drinking the highest volume of coffee (P95th), the values were lower than 10^−4^, meaning that there is a potential risk in the consumption of coffee in Costa Rica due to AA intake. The MOE results also indicated that there is a potential carcinogenic risk of AA intake due to coffee consumption. Therefore, some risk mitigation actions should be considered. In the literature, it was found that AA concentration decreased during the storage periods from (12 to 80) % in (6–12) months at room temperature for ground and soluble coffee [42,43,44,45]. However, coffee includes more than 800 volatiles, and storage affects the volatile profile of coffee [46]. So, optimization should be performed to find the storage time that guarantees the maximum AA loss and conservation of the highest concentration of volatile compounds. Another action could be the use of asparaginase. It has been reported that AA concentration decreased from 59 to 75% by using asparaginase, while there were no changes in major phenolic compounds such as chlorogenic and caffeic acids [47,48]. The other action that can be taken is to optimize the roasting process [49,50,51]. The disadvantage of this action is that any changes in the roasting process may also affect the properties and consumer acceptability of the product. Therefore, these mitigation alternatives must be weighed so that risk management actions can be applied.

## 5. Conclusions

This study aimed to evaluate the occurrence of AA in roasted coffee products commercially sold in retail markets of Costa Rica and to develop a probabilistic exposure assessment model to assess the potential human health risk of AA exposure among coffee consumers in this country. The results indicated that the average AA concentration in coffee was 110.29 ± 151.61 µg kg^−1^. Regarding the DE results, the female minors had a higher AA intake compared to the other subpopulations, because of their low BW. Even though no neurotoxicity risk for AA intake due to coffee consumption in Costa Rica was found, a potential carcinogenic risk was observed based on MOE and ILCR results. Considering the above, additional treatments might be required to reduce the AA intake due to coffee consumption in Costa Rica; thus, risk management strategies should be considered.

## Figures and Tables

**Table 1:** Summary of selected validation parameters for GC-MS determination of AA in grounded roasted coffee.

Linearity (as Coefficient of Determination)	LOD ^1^	LOQ ^2^	Recovery (Spiking at 0.3 μg mL^−1^)	Recovery (FAPAS^®^ Quality Control Material)
	μg kg^−1^		%	
0.992–0.999	25.00	75.75	85–90	72–122

^1^ LOD is the Limit of detection. ^2^ LOQ is the Limit of quantification.

**Table 2:** Occurrence (%) and concentration of acrylamide (µg kg^−1^) in Arabica coffee products commercially sold in Costa Rican retail markets, according to coffee type (*n* = 110).

Type of Coffee	Coffee Samples			Concentration of AA (µg kg^−1^) ^2^		
	*n*	*x* ≥ LOD ^1^				
		*n*	%	Mean ± SD	Minimum	Maximum
Light roast pure	7	5	71.4	192.41 ± 143.96	95.16	446.14
Medium roast pure	49	28	57.1	138.38 ± 64.19	33.41	292.20
Dark roast pure	30	21	70.0	201.23 ± 178.95	78.42	696.18
Total of pure roasted	86	54	62.8	167.83 ± 129.25	33.41	696.18
Torrefacto	12	0	0.0	NA ^3^	NA	NA
Soluble	12	8	66.7	383.61 ± 192.67	186.28	759.59
Total	110	62	56.4	195.67 ± 155.22	33.41	759.59

^1^ Limit of detection (LOD) ≤ 25 µg kg^−1^. ^2^ Values were calculated based on the number of samples above the limit of detection. ^3^ NA: not applicable.

**Table 3:** The daily dietary exposure to acrylamide (µg kg^−1^ BW per day) due to Arabica coffee consumption for different urban subpopulations in Costa Rica.

Population	Mean			P5th			P50th			P95th		
	LB ^1^	MB ^2^	UB ^3^	LB	MB	UB	LB	MB	UB	LB	MB	UB
Male adults	0.025	0.027	0.028	0.001	0.003	0.004	0.0139	0.0156	0.0174	0.092	0.091	0.090
Female adults	0.024	0.025	0.026	0.001	0.002	0.004	0.0139	0.0156	0.0173	0.085	0.083	0.083
Male minors	0.027	0.028	0.030	0.001	0.002	0.004	0.0124	0.0138	0.0152	0.088	0.090	0.092
Female minors	0.079	0.083	0.087	0.001	0.002	0.004	0.0131	0.0148	0.0164	0.142	0.147	0.153

^1^ LB: lower bound approach. ^2^ MB: middle-bound approach. ^3^ UB: upper-bound approach.

**Table 4:** (**A**) Risk characterization of acrylamide neurotoxicity, using margin of exposure (MOE), due to Arabica coffee consumption for different urban subpopulations in Costa Rica. (**B**) Risk characterization of acrylamide carcinogenecity, using margin of exposure (MOE), due to Arabica coffee consumption for different urban subpopulations in Costa Rica.

(**A**)												
**Population**	**Mean**			**P5th**			**P50th**			**P95th**		
	**LB ^1^**	**MB ^2^**	**UB ^3^**	**LB**	**MB**	**UB**	**LB**	**MB**	**UB**	**LB**	**MB**	**UB**
Male adults	16,991	16,175	15,433	4291	4768	4711	29,995	27,509	24,676	467,984	174,195	113,343
Female adults	18,214	17,340	16,544	4566	5112	5207	30,100	27,614	24,741	477,203	171,728	112,733
Male minors	15,944	15,179	14,482	4265	4729	4652	33,634	31,121	28,213	506,062	183,678	117,571
Female minors	5450	5188	4950	2512	2881	2817	31,725	28,995	26,250	495,151	189,686	122,465
(**B**)												
**Population**	**Mean**			**P5th**			**P50th**			**P95th**		
	**LB ^1^**	**MB ^2^**	**UB ^3^**	**LB**	**MB**	**UB**	**LB**	**MB**	**UB**	**LB**	**MB**	**UB**
Male adults	6717	6394	6101	1696	1885	1863	11,858	10,876	9756	185,017	68,868	44,810
Female adults	7201	6855	6541	1805	2021	2059	11,900	10,917	9781	188,661	67,893	44,569
Male minors	6304	6001	5726	1686	1867	1839	13,297	12,304	11,154	200,071	72,617	46,481
Female minors	2155	2051	1957	993	1139	1114	12,543	11,463	10,378	195,757	74,992	48,317

^1^ LB: lower bound approach. ^2^ MB: middle-bound approach. ^3^ UB: upper-bound approach.

**Table 5:** Incremental lifetime cancer risk (ILCR) for Costa Rican consumers due to AA intake from coffee.

Population	Mean			P5th			P50th			P95th		
	LB ^1^	MB ^2^	UB ^3^	LB	MB	UB	LB	MB	UB	LB	MB	UB
Male adults	1.6 × 10^−5^	1.7 × 10^−5^	1.8 × 10^−5^	4.7 × 10^−7^	1.5 × 10^−6^	2.4 × 10^−6^	8.5 × 10^−6^	9.6 × 10^−6^	1.1 × 10^−5^	7.7 × 10^−5^	7.8 × 10^−5^	7.5 × 10^−5^
Female adults	1.5 × 10^−5^	1.6 × 10^−5^	1.7 × 10^−5^	4.5 × 10^−7^	1.5 × 10^−6^	2.3 × 10^−6^	8.5 × 10^−6^	9.5 × 10^−6^	1.1 × 10^−5^	7.0 × 10^−5^	6.7 × 10^−5^	6.8 × 10^−5^
Male minors	1.8 × 10^−5^	1.9 × 10^−5^	1.9 × 10^−5^	4.3 × 10^−7^	1.4 × 10^−6^	2.9 × 10^−6^	7.6 × 10^−6^	8.5 × 10^−6^	9.3 × 10^−6^	8.5 × 10^−5^	8.6 × 10^−5^	8.7 × 10^−5^
Female minors	3.5 × 10^−5^	3.9 × 10^−5^	3.5 × 10^−5^	4.4 × 10^−7^	1.2 × 10^−6^	1.8 × 10^−6^	8.1 × 10^−6^	8.0 × 10^−6^	8.8 × 10^−6^	8.7 × 10^−5^	1.5 × 10^−4^	1.5 × 10^−4^

^1^ LB: lower bound approach. ^2^ MB: middle-bound approach. ^3^ UB: upper-bound approach.

## Data Availability

The original contributions presented in the study are included in the article; further inquiries can be directed to the corresponding author.

## Abbreviations

The following abbreviations are used in this manuscript:

AA	Acrylamide
BDML_10_	Benchmark lower dose level
BW	Body Weight
DE	Dietary exposure
ELANS	Latin American Study of Nutrition and Health
IARC	International Agency for Research on Cancer
ILCR	Incremental lifetime cancer risk
LB	Lower bound
LOD	Limit of detection
LOQ	Limit of quantification
MB	Middle bound
MOE	Margin of exposure
MPL	Maximum permitted level
UB	Upper bound
